# From Stable Traits to Dynamic Mechanisms: An Integrative Conceptual Model of Personality and Criminogenic Cognitions in the Incarcerated Population

**DOI:** 10.3390/bs16091604

**Published:** 2026-09-09

**Authors:** Cornelia Rada

**Affiliations:** Biomedical Department, Francisc I. Rainer Institute of Anthropology, Romanian Academy, Academy House, 13 September Avenue, No. 13, 5th District, 050711 Bucharest, Romania; corneliarada@yahoo.com

**Keywords:** personality traits, criminogenic cognitions, incarcerated population, HEXACO, Dark Triad, self-efficacy, self-control

## Abstract

Criminal behavior has been explained through multiple theoretical frameworks developed independently, including general personality models (FPI, Big Five, HEXACO), maladaptive configurations (Dark Triad/Tetrad, psychopathy), and criminogenic cognitions. However, integrated analysis of this literature suggests the existence of a multilevel architecture of antisocial vulnerability rather than entirely distinct, non-communicating mechanisms. This narrative integrative review synthesizes empirical evidence regarding these frameworks in incarcerated populations, drawing predominantly on literature indexed in Web of Science and Scopus, supplemented by Google Scholar and snowballing searches, as detailed in the Methods section. The analysis identifies a convergent dispositional core—characterized by elevated aggression, emotional excitability, and psychological strain, alongside reduced conscientiousness, agreeableness, honesty–humility, and social integration—that may exert its influence on behavior partly through dynamic mechanisms such as self-efficacy and self-control, together with the more proximal influence of criminogenic cognitions, expressed differentially through maladaptive personality configurations. The persistent heterogeneity of offender profiles and conceptual convergences across models support this same multilevel structure. The findings propose a three-level integrative conceptual model that explicitly links these previously fragmented studies, with implications for directing correctional interventions toward dynamic mechanisms rather than solely toward stable personality traits.

## 1. Introduction

Criminal behavior results from a complex interaction between biological, psychological, and social factors, being influenced both by individual characteristics and by the family, educational, and socio-cultural context in which the person develops (Bonta & Andrews, 2017; Farrington et al., 2016; Glenn & Raine, 2014). Among individual factors, personality occupies a central place in explaining the variability of antisocial behavior, with numerous studies demonstrating the association of specific traits with the onset, persistence, and severity of delinquent conduct (Miller & Lynam, 2001; S. E. Jones et al., 2011; Vize et al., 2019). Although this role is widely recognized, the conceptualization and assessment of personality have evolved considerably, reflecting the complexity of the construct and the need to capture complementary psychological dimensions (Costa & McCrae, 1992; Anglim & O’Connor, 2019; Tharshini et al., 2021).

In the specialized literature, the assessment of offender populations has been conducted through distinct conceptual frameworks, each offering a particular perspective on individual psychological functioning. The Freiburg Personality Inventory (FPI) highlights classic personality dimensions and features of psychosocial adaptation (Fahrenberg et al., 2001); the Big Five model describes the general structure of personality traits (Costa & McCrae, 1992); and the HEXACO model extends this perspective by including the Honesty–Humility dimension, considered relevant for understanding antisocial behaviors and moral conduct (Ashton & Lee, 2007). At the same time, models centered on dark personality traits and psychopathy investigate maladaptive characteristics associated with manipulation, impulsivity, lack of empathy, and interpersonal exploitation (Paulhus & Williams, 2002; Furnham et al., 2013; Hare & Neumann, 2019), while theories of criminogenic cognitions complement this perspective through the analysis of beliefs, cognitive justifications, and thinking patterns that facilitate and maintain criminal behavior (Tangney et al., 2012; Walters, 2022; Bonta & Andrews, 2017). These models do not represent competing perspectives but complementary approaches, describing different levels of psychological functioning involved in the emergence and maintenance of criminal behavior.

Numerous empirical studies and meta-analyses confirm that personality traits and cognitive factors are significant predictors of criminal behavior, being involved in the initiation, persistence, and recidivism of antisocial conduct, as well as in the response to psychological interventions (Miller & Lynam, 2001; S. E. Jones et al., 2011; Vize et al., 2019; Walters, 2022). Reported findings remain heterogeneous, however, as each model investigates different constructs, uses specific psychometric instruments, and emphasizes particular explanatory mechanisms (Anglim & O’Connor, 2019; Tharshini et al., 2021). Consequently, the literature remains fragmented both conceptually and methodologically: most studies address the general dimensions of personality, maladaptive traits, or criminogenic cognitions separately, without systematically examining the complementary relationships among them (Tharshini et al., 2021; Hampejs et al., 2025). This independent development has limited the construction of an integrative explanatory framework, and the psychological profile of incarcerated individuals continues to be described through findings dispersed across different models and instruments, which complicates the comparison of conclusions and the identification of convergently reported characteristics in the empirical literature.

Building on this gap, the present study aims to provide an integrative synthesis of the empirical evidence on personality traits and criminogenic cognitions associated with incarcerated populations, using a narrative integrative review approach. The analysis is based predominantly on empirical studies published in journals indexed in Web of Science and Scopus, supplemented, where relevant, by meta-analyses and systematic reviews, and is organized around five conceptual frameworks: FPI, Big Five, HEXACO, Dark Triad/Dark Tetrad and psychopathy, and criminogenic cognitions. By integrating these perspectives into a common conceptual model, the present review aims to provide a coherent picture of the convergences and complementarities among general personality traits, maladaptive configurations, and criminogenic cognitions, facilitating an integrated interpretation of the psychological profile associated with criminal behavior.

Beyond broad-band personality dimensions, incarcerated populations have also been characterized through maladaptive personality configurations—principally the Dark Triad (Machiavellianism, narcissism, and subclinical psychopathy; Paulhus & Williams, 2002) and, in more recent formulations, the Dark Tetrad, which adds everyday sadism (D. N. Jones & Paulhus, 2014)—and through psychopathy examined as a distinct, more severe construct consistently associated with instrumental violence and persistent recidivism (Hare & Neumann, 2019). These maladaptive configurations are theorized to occupy an intermediate position between the broad dispositional traits captured by the Big Five and HEXACO models and the proximal cognitive processes addressed by the criminogenic-cognitions literature—criminal thinking styles, cognitive distortions, and neutralization techniques that justify and sustain offending behavior (Tangney et al., 2012; Walters, 2022). Because these three bodies of literature have developed largely along parallel, non-communicating lines, integrating them into a single explanatory account requires examining not only the independent empirical support for each, but also the points of conceptual overlap and complementarity among them—the central objective pursued in the present review.

Given the non-interventional, associative nature of the present synthesis, the guiding question is formulated not in PICO terms—a structure developed for intervention studies with an explicit comparator arm—but in the corresponding PEO format (Population, Exposure, Outcome), more appropriate to observational and correlational evidence: among incarcerated or offender populations (Population), how are general personality traits, maladaptive personality configurations, and criminogenic cognitions (Exposure constructs, examined comparatively across the five conceptual frameworks reviewed) associated with criminal behavior, aggression, and recidivism (Outcome)?

## 2. Methodology

### 2.1. Study Design

The present work employs a narrative integrative review design (narrative/critical review), aimed at the conceptual synthesis of the literature on personality and criminogenic cognitions in incarcerated populations. This methodological choice was preferred over a systematic review or a scoping review structured according to PRISMA-ScR (Tricco et al., 2018), because the central objective of the study is not to quantify or critically evaluate the methodological quality of individual studies, but rather to organize, integrate, and conceptually interpret a body of literature fragmented across multiple models concerning personality and criminogenic cognitions, each with distinct terminologies, instruments, and research traditions. Narrative integrative reviews represent an established approach frequently used in psychology and criminology for this type of objective, allowing the construction of synthesizing conceptual models that go beyond the boundaries of a single theoretical framework (Grant & Booth, 2009; Baumeister & Leary, 1997).

### 2.2. Literature Search Strategy

The identification of relevant literature was carried out through searches in major academic databases—Google Scholar, Scopus, and Web of Science (Core Collection)—supplemented by snowballing strategies (forward and backward citation tracking), by tracing the references of relevant articles and the works that cite these studies.

The search terms included combinations of the following keywords: “personality,” “prisoners,” “incarcerated population,” “FPI,” “Freiburg Personality Inventory,” “Big Five,” “HEXACO,” “Dark Triad,” “Dark Tetrad,” “psychopathy,” “criminogenic cognitions,” and “criminal thinking styles,” used both individually and in multiple combinations (Boolean operators AND/OR), in order to capture the terminological variability specific to the personality psychology and criminology literature. Searches were limited to sources in English, with the exception of a small number of foundational works originally published in German (concerning the Freiburg Personality Inventory), included for their historical and conceptual relevance and cited from available translations/adaptations.

The time frame analyzed was, with some justified exceptions, 1990–May 2026, in order to cover both the foundational developments of the main personality models used in forensic contexts and the contemporary literature. A limited number of works predating this interval were also included (Hesener & Hilse, 1983; Kury, 1983; Ortmann, 1988), essential for documenting the earliest applications of the FPI in prison populations, in the absence of which the historical development of this line of research could not be reconstructed.

### 2.3. Inclusion Criteria

Empirical studies, peer-reviewed articles, theoretical works, and relevant academic volumes investigating at least one of the following constructs were included: the Freiburg Personality Inventory (FPI), the Big Five model, the HEXACO model, Dark Triad/Dark Tetrad, psychopathy, and criminogenic cognitions/criminal thinking styles. Priority was given to studies conducted on incarcerated or offender populations (convicted inmates or individuals in pretrial detention); a limited number of studies on non-incarcerated populations were included exclusively for conceptual clarification, instrument definition, or documentation of general psychological mechanisms (e.g., the neurobiological basis of psychopathic traits) directly relevant to interpreting findings from the incarcerated population.

### 2.4. Exclusion Criteria

The following were excluded: studies without direct relevance to the relationship between personality, cognitions, and criminal behavior; non-empirical publications lacking a clear theoretical foundation (opinions, editorials without scientific analysis); studies on exclusively non-incarcerated populations that did not meet the exception condition mentioned above; duplicate sources; and studies that did not present explicit links between the analyzed constructs and criminal behavior or delinquency.

### 2.5. Selection Process and Data Organization

The selection and analysis of the literature were carried out by a single author, an option consistent with the narrative-integrative nature of the review, but one that constitutes an acknowledged methodological limitation: the absence of independent inter-rater evaluation (dual screening) entails a risk of subjectivity in the selection and interpretation of sources—a risk that classic systematic designs or structured scoping reviews reduce through standardized double-verification procedures. For this reason, the present review does not aim for the exhaustiveness of a systematic review but rather constitutes an argued conceptual synthesis of the literature identified as relevant through the procedure described above.

The final corpus of the review consists of 109 unique sources, distributed across the five theoretical frameworks analyzed (FPI, Big Five, HEXACO, Dark Triad/Tetrad and psychopathy, and criminogenic cognitions). Data extracted from the included literature were organized thematically, tracking for each source: the main theoretical framework investigated, the population and sample studied, the assessment instruments used, and the main findings reported in relation to criminal behavior. This organization allowed for the identification of conceptual convergences and divergences among models, as well as psychological patterns consistently reported in the incarcerated population, synthesized in the integrative conceptual model presented in Section 3 and discussed comparatively in the Section 5.

**Figure 1:**
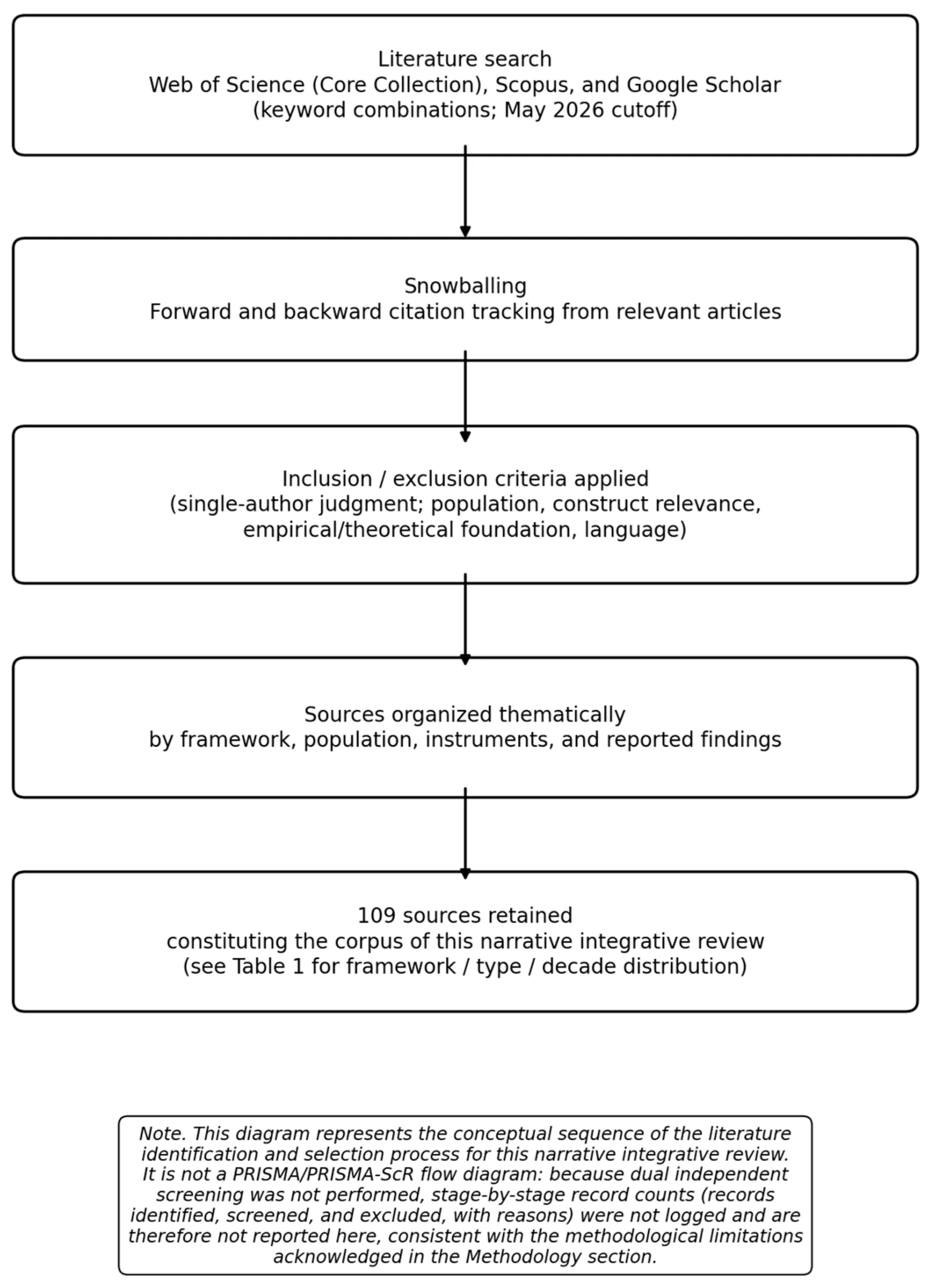
Conceptual overview of the literature identification and selection process.

To make the composition of this corpus transparent, Table 1 reports the distribution of the 109 included sources across the five theoretical frameworks (plus a cross-cutting/general/methodological category comprising sources not specific to a single framework, e.g., general criminology, review methodology, and psychometric instrument sources shared across frameworks), broken down by source type. Regarding the overlap between the analytic corpus and the reference list raised in review: because this is a narrative integrative review rather than a systematic review with a separate background/introduction citation pool, sources cited for conceptual or historical framing (e.g., foundational instrument manuals, general criminological theory) are drawn from the same corpus as those analyzed for empirical findings; Table 1 shows that a substantial proportion of this corpus (27 of 109 sources, 24.8%) falls into this cross-cutting/general category rather than being framework-specific empirical evidence, which we report explicitly here to allow the reader to judge the balance between framework-specific empirical support and general/theoretical framing. Regarding temporal distribution, sources predate 1990 in 5 cases (4.6%, retained for documenting the historical origins of the FPI, as noted above), fall in the 1990s in 3 cases (2.8%), the 2000s in 17 cases (15.6%), the 2010s in 46 cases (42.2%), and 2020 onward in 38 cases (34.9%), reflecting the deliberate emphasis on contemporary literature alongside foundational sources described in the Literature Search Strategy above. The reference list accompanying this article contains 111 entries in total: the 109 sources constituting the analytic corpus reported in Table 1, plus two additional sources (Shields & Simourd, 1991; Simourd, 1997) cited exclusively to define a comparison instrument (the Criminal Sentiments Scale—Modified) mentioned in Section 4.5, consistent with the inclusion-criteria exception, stated above, for sources included solely for instrument definition rather than as part of the synthesized evidence base (Figure 1).

### 2.6. Positioning Relative to Systematic Review Standards

Because several elements of the search and selection procedure described above (explicit databases, search terms, inclusion/exclusion criteria) resemble the apparatus of a systematic or scoping review, it is necessary to state explicitly which methodological components typically required by such designs (e.g., PRISMA-ScR; Tricco et al., 2018) were adopted in the present work and which were not, together with the rationale for each omission, in order to prevent the reader from interpreting this review as methodologically equivalent to a systematic or scoping review. Adopted: an explicitly described search strategy across named databases; explicit inclusion and exclusion criteria; an explicit and justified time frame. Not adopted, with rationale: (a) a PRISMA-ScR flow diagram and exact stage-by-stage counts of records identified, screened, and excluded were not produced, because the objective of the work is conceptual integration of a fragmented literature rather than exhaustive mapping of a bounded evidence base, and because single-author screening would render such counts methodologically uninformative in the absence of independent verification; (b) dual, independent screening and the corresponding inter-rater agreement statistic were not implemented, given the single-author design acknowledged below as a limitation; (c) no formal study-quality or risk-of-bias appraisal (e.g., MMAT, Newcastle-Ottawa Scale) was conducted, since the corpus spans five conceptual frameworks assessed through heterogeneous designs and instruments for which a single common appraisal tool is not directly applicable—this is acknowledged as a limitation of the present synthesis rather than a deliberate methodological choice; (d) no meta-analytic pooling of effect sizes or structural equation modeling was performed, as the aim of the work is the conceptual convergence, not the statistical aggregation, of findings obtained across heterogeneous populations, instruments, and metrics. The three-level model proposed in Section 3 should accordingly be read in light of these boundaries.

### 2.7. Limitations of the Methodological Design

The narrative nature of the present review entails limitations that must be explicitly acknowledged. In the absence of a systematic PRISMA-ScR procedure, it is not possible to report exact figures regarding the number of records identified, screened, and excluded at each stage, and the single-author selection does not allow for the calculation of an inter-rater agreement index. Consequently, the results of this work should be interpreted as an argued conceptual synthesis of the literature considered relevant, rather than as an exhaustive and fully reproducible mapping of the field. Future research could revisit this topic through a scoping review structured according to PRISMA-ScR, with dual selection and full reporting of the screening process, in order to validate and extend the conceptual convergences identified in the present work.

## 3. Integrative Conceptual Model of the Psychological Functioning Associated with Criminal Behavior

### 3.1. Foundations of the Conceptual Model

Building on the conceptual and methodological fragmentation documented above, the present study proposes an integrative conceptual model that organizes the convergences identified among the analyzed studies into a hierarchical structure comprising three complementary psychological levels: (1) the dispositional level of personality, (2) the level of maladaptive configurations, and (3) the level of proximal cognitive mechanisms. The model does not aim to replace existing approaches, but rather to integrate them into a common explanatory framework, in which criminal behavior is understood as the result of a dynamic interaction among stable individual predispositions, maladaptive personality organizations, and cognitive processes that facilitate or justify the manifestation of antisocial behavior.

### 3.2. Conceptual Level I—General Personality Traits

The first level corresponds to the dispositional structure of personality, represented in this review by the FPI, Big Five, and HEXACO models (Costa & McCrae, 1992; Ashton & Lee, 2007). Combinations such as elevated impulsivity, reduced conscientiousness, low agreeableness, emotional instability, or reduced levels of Honesty–Humility are repeatedly associated with antisocial behavior, functioning as distal dispositional factors that increase individual vulnerability without directly determining criminal conduct (Vize et al., 2019).

### 3.3. Conceptual Level II—Maladaptive Personality Configurations

The second level is represented by the Dark Triad, Dark Tetrad, and psychopathy constructs, which describe characteristics associated with persistent antisocial functioning—interpersonal manipulation, lack of empathy, egocentrism, impulsivity, and shallow affect (Paulhus & Williams, 2002; Hare & Neumann, 2019). These traits show more direct associations with violence, aggression, and recidivism compared to general personality dimensions, representing the intermediate level of psychological vulnerability (Geerlings et al., 2020; Gillespie et al., 2023). Psychopathy occupies a central role at this level, integrating affective, interpersonal, and behavioral dimensions. To avoid conceptual ambiguity, psychopathy is treated throughout this review as a dimensional personality construct—a continuously distributed pattern of interpersonal, affective, and behavioral traits (Patrick et al., 2009), detailed in Section 4.4—rather than as a categorical clinical diagnosis; where cited studies assess it through categorical or diagnostic procedures, this is noted explicitly at the point of citation.

### 3.4. Conceptual Level III—Criminogenic Cognitions

The third level corresponds to proximal cognitive processes—criminal thinking styles, cognitive distortions, neutralization mechanisms, and belief systems that justify or sustain criminal behavior. Unlike stable personality traits, criminogenic cognitions mediate the relationship between individual predispositions and observable conduct, being the component closest to the manifestation of behavior and a central target of interventions aimed at reducing recidivism (Tangney et al., 2012; Walters, 2022).

#### Integration of the Psychological Levels

The proposed conceptual model integrates the three levels into a unified perspective, conceptualizing criminal behavior as the outcome of a multilevel psychological cascade, in which distal personality influences interact with maladaptive structures and proximal cognitive processes, without presupposing unidirectional causal relationships. The ordering of the three levels is conceptual rather than developmental or empirically established: it reflects an organizing distinction based on proximity to manifest behavior, not a demonstrated temporal or causal sequence, and it does not preclude direct or reciprocal relationships between constructs assigned to different levels—for instance, between psychopathy and general personality traits, or between criminogenic cognitions and psychopathy. This model provides the organizing framework for the following chapters, underpinning the comparative interpretation of the five perspectives investigated. It should be stated explicitly that this three-level structure constitutes an interpretive synthesis derived from a single-author narrative review of independently conducted primary studies, and not a structure statistically derived from meta-analysis, structural equation modeling, or network analysis. Accordingly, the levels and their interrelations proposed below should be read as an organizing heuristic supported by convergent findings across the literature examined, rather than as an empirically validated causal architecture; direct verification through a single integrated design remains, as discussed in Section 5, an open avenue for future research.

## 4. Literature Analysis Across the Five Conceptual Frameworks

### 4.1. Personality Dimensions in the Incarcerated Population Assessed Through the Freiburg Personality Inventory (FPI)

The Freiburg Personality Inventory (FPI), developed by Fahrenberg, Selg, and Hampel and first published in 1970, represents one of the earliest multidimensional personality assessment instruments developed within continental European psychology (Fahrenberg et al., 2001). Developed within the German-speaking psychological tradition, the instrument assesses dimensions relevant to psychological and social adjustment, such as aggressiveness, emotional excitability, social inhibition, interpersonal orientation, and affective stability—traits confirmed by the systematic literature as individual factors relevant to criminality (Tharshini et al., 2021)—and has been used in criminological and forensic research to describe the psychological profile of individuals involved in criminal behavior and to analyze adaptation to the prison environment.

The revised form, the FPI-R, contains 138 items with a dichotomous response format (“True”/”False”) and assesses twelve personality dimensions: life satisfaction, social orientation, achievement orientation, inhibition, emotional excitability/irritability, aggressiveness, psychophysiological tension, somatic complaints, health concerns, sincerity/openness, extraversion, and emotionality/neuroticism (Fahrenberg et al., 2001).

Although the use of the FPI in the international criminological literature has declined considerably over the past decade, the instrument continues to be used mainly in research conducted in German-speaking countries (Germany, Austria, Switzerland), predominantly in psychological and psychiatric-forensic contexts, focusing on characterizing the personality profiles of incarcerated individuals, evaluating the psychometric properties of the instrument in the prison environment, and examining the relationship between personality traits, institutional adaptation, and violent behavior.

#### 4.1.1. Studies on Offender Populations

The earliest studies using the FPI in offender populations revealed a psychological profile distinct from that of the general population. In a study of incarcerated juvenile delinquents, Hesener and Hilse (1983) found significantly higher scores on nervousness, spontaneous aggressiveness, irritability, depressiveness, and neuroticism, together with lower levels of social orientation and emotional balance, interpreted as increased vulnerability to affective instability and difficulties in interpersonal integration. The authors drew attention to the influence of the prison context on self-report responses—an observation extended by Kury (1983), who investigated response distortion tendencies among young inmates using the adolescent version of the FPI. The results showed that participants who were informed that the assessment would be included in their prison file displayed favorable self-presentation, particularly by minimizing aggressive behaviors; explicit instructions to reduce faking diminished, without fully eliminating, these effects, highlighting the vulnerability of self-report instruments to institutional context.

#### 4.1.2. Validity of the FPI-R in Assessing Prison Populations

An important milestone is the study by Hosser et al. (2008), who evaluated the psychometric properties of the FPI-R in a sample of 775 inmates from the German prison system. The results support satisfactory validity and reliability in a forensic context, although certain scales showed altered factorial properties compared to the general population; the identified profile was characterized by elevated aggressiveness, excitability, and psychological strain, associated with reduced social orientation. The authors emphasized that the institutional environment may influence responses, recommending that results be interpreted in light of the specific characteristics of the population assessed.

Although the FPI-R was designed to assess relatively stable traits, Fichter and Quadflieg (2000) showed, in a study of patients undergoing intensive inpatient therapy, that dimensions related to emotional control, self-perception, and the management of psychological difficulties can change as a result of therapeutic intervention. Although not conducted on offender populations, the study provides evidence for the sensitivity of certain FPI-R dimensions to change and qualifies the interpretation of personality traits as entirely stable variables.

#### 4.1.3. The FPI and Adaptation to the Prison Environment

Ortmann (1988) conceptually analyzed the role of personality traits in the process of prisonization, arguing that emotional instability, aggressiveness, and excitability influence both vulnerability to criminal behavior and the way incarcerated individuals respond to the constraints of the prison environment; impulsivity, difficulties in emotional regulation, and deficient social integration may foster poor adjustment to detention. Although theoretical, this work provided a benchmark for subsequent research on the interaction between personality and the institutional environment.

This perspective is empirically supported by Ille et al. (2005), who, in a sample of 128 individuals undergoing psychiatric-forensic evaluation in Germany (assessed with the FPI-R and the Freiburg Aggression Factors), identified three distinct profiles through cluster analysis: a dominant profile characterized by elevated aggressiveness and excitability together with emotional instability; a profile marked by inhibition and reduced social integration; and a profile close to non-deviant functioning. These findings highlight the heterogeneity of offender populations and the ability of the FPI-R to differentiate subgroups with clinical and criminological relevance, including between first-time and repeat offenders.

#### 4.1.4. Recent Research and Implications for Psychological Assessment

Volf (2015) analyzed the applicability of the FPI-R in the prison environment, identifying a profile characterized by heightened emotional reactivity, impulsivity, and difficulties in behavioral regulation, associated with reduced social adaptation and less effective coping strategies. The author notes that the instrument is used mainly for descriptive purposes, to differentiate subgroups, its predictive value being more limited, and mentions the limitations arising from the prison context and the absence of validated predictive models for criminal risk.

Convergence with specialized risk assessment instruments is illustrated by Endrass et al. (2008), who, in a sample of 113 Swiss inmates assessed, among other instruments, with the PCL-R, identified associations between antisocial personality traits and institutional violent incidents (with higher predictive capacity for verbal aggression than for physical aggression). The profile described—impulsivity, behavioral instability, difficulties in controlling aggression—overlaps conceptually with the dimensions of aggressiveness, excitability, and emotional instability captured by the FPI-R, although the Freiburg instrument was not used directly in this study.

Contemporary confirmation is provided by Rada et al. (2025a), who, in a sample of 857 incarcerated individuals from Romanian prisons, tested, using the FPI, the Self-Efficacy Scale, and the COPE Inventory, a moderated mediation model: self-efficacy mediates the relationship between personality traits and coping mechanisms, influencing adaptation to detention. The participants’ profile was characterized by elevated aggressiveness, psychological strain, health concerns, and emotionality, with variations according to age, sex, and length of detention—a finding that highlights both the psychological heterogeneity of the prison population and the importance of integrating cognitive and motivational variables in assessing adaptation to incarceration.

The available literature on the FPI converges on a personality profile defined by elevated aggressiveness, emotional excitability, and psychological strain, together with difficulties in impulse regulation and reduced social integration (Hesener & Hilse, 1983; Hosser et al., 2008; Ille et al., 2005; Volf, 2015; Rada et al., 2025a), summarized in Table 2.

The early dimensions identified through the FPI—aggressiveness, emotional reactivity, and social integration—are reflected, under different labels and conceptualizations, in contemporary personality assessment models: the Big Five (S. E. Jones et al., 2011), the MMPI-2-RF and MMPI-3 (Sellbom et al., 2022), the Personality Assessment Inventory (Douglas et al., 2001), and the PID-5 (Dunne et al., 2021). This continuity supports a line of conceptual development linking classic research to current approaches. From this perspective, the FPI can be regarded as the starting point of a conceptual evolution toward current structural models, particularly the Big Five, to which the following chapter is dedicated.

A note on the evidentiary status of the sources summarized in Table 2 is warranted. Hesener and Hilse (1983) assessed *N* = 166 incarcerated male juvenile delinquents using the FPI, drawn from two facilities (Adelsheim, *n* = 96; Hameln-Tündern, *n* = 70) and analyzed as a combined sample (Gesamtpopulation) relative to the male normative sample aged 15–30; a central finding of their study was that anonymous versus name-identified administration of the FPI (Offenheit vs. Nichtoffenheit) significantly affected participants’ response patterns, a methodological point relevant to the interpretation of self-report personality data obtained in custodial settings more generally. Hosser et al. (2008), in a psychometric validity study of *N* = 775 male German incarcerated juveniles (Jugendstrafvollzug) assessed at intake, report that inmates’ scores on the Aggressiveness, Inhibition, and Social Orientation scales deviate from age-specific norms in a direction contrary to expectation (Hosser et al., 2008); the published abstract does not specify the direction of this deviation, and full-text verification was not possible within the scope of this review, so this finding is reported here as evidence of a documented deviation from age norms on these three scales, not as confirmation of the elevated pattern summarized in Table 2. Volf (2015) is a master’s thesis (Charles University, Prague, unpublished) rather than a peer-reviewed journal article; it is retained here, at the author’s decision, in the absence of an equivalent published source on the FPI in a comparable prison population, and should be weighted accordingly by the reader.

### 4.2. Personality Dimensions in the Incarcerated Population Through the Big Five Model

The Big Five model (Five-Factor Model—FFM) represents one of the most validated dimensional models of personality and a central theoretical framework for explaining the relationship between personality traits and criminal behavior. Developed by Costa and McCrae (1992), it structures personality into five major dimensions: extraversion, agreeableness, conscientiousness, neuroticism, and openness to experience. Whereas clinico-descriptive instruments such as the FPI enabled the first systematic assessments of personality in offender populations, the Big Five provided the dimensional framework necessary for cross-cultural comparability and the integration of data into explanatory models of antisocial behavior, moving beyond traditional categorical classifications (“offender” vs. “non-offender”) in favor of an analysis of continuous individual variation (Costa & McCrae, 1992; Miller & Lynam, 2001).

#### 4.2.1. Meta-Analytic Foundations and the Externalizing Spectrum

The meta-analysis by Miller and Lynam (2001) integrates four theoretical models—PEN (Eysenck & Eysenck, 1975), Positive Emotionality–Negative Emotionality–Constraint (Tellegen, 1985; Patrick et al., 2013), the Big Five, and Cloninger’s psychobiological model (temperament and character; Cloninger et al., 1993)—and identifies a common core of antisocial behavior: low self-control and conscientiousness, elevated negative affectivity, high impulsivity, and sensation seeking, accompanied by reduced prosocial behavior. Low agreeableness and conscientiousness are consistently confirmed, in subsequent literature, as the strongest predictors of aggression and delinquency (S. E. Jones et al., 2011; Shimotsukasa et al., 2019; Anglim & O’Connor, 2019), supporting the notion of a central self-regulation deficit.

This configuration is embedded within a broader externalizing spectrum, characterized by interpersonal antagonism, impulsivity, behavioral disinhibition, and self-regulation deficits. The meta-analysis by Vize et al. (2019) identifies antagonism and disinhibition as the common psychological core of antisocial behaviors, while Sleep et al. (2018) show that maladaptive traits from the DSM-5 model overlap significantly with Big Five profiles associated with criminality, particularly through elevated antagonism and low agreeableness/conscientiousness. These findings support the existence of a unitary externalizing spectrum—aggression, substance use, risk-taking behaviors, recidivism (DeLisi, 2016)—defined by impulsivity and low frustration tolerance.

#### 4.2.2. Differentiated Profiles by Type of Criminality

Different forms of criminality are associated with distinct profiles: violent crime with antagonism, impulsivity, elevated neuroticism, and hostility, while economic crime is associated with low conscientiousness and inhibitory control deficits (S. E. Jones et al., 2011). In sexual delinquency, profiles are heterogeneous—ranging from elevated neuroticism and introversion to antagonism and empathy deficits, depending on offense typology (DeLisi, 2016). A study of Japanese inmates (*N* = 645) compared with the general population (*N* = 4546) confirms this differentiation: violent offenders show lower agreeableness and higher extraversion, thieves show reduced conscientiousness, and drug offenders show higher levels of extraversion and openness (Shimotsukasa et al., 2019).

Neuroticism is positively associated with antisocial behavior, but its effects vary contextually: Jahnke et al. (2019) show that individuals with a sexual interest in minors present elevated neuroticism and introversion but also increased conscientiousness—contrary to classic profiles reported in the prison literature, which suggests the influence of impression management and the institutional environment on reported profiles (Shimotsukasa et al., 2019; Jahnke et al., 2019). These variations indicate that, although low agreeableness and conscientiousness remain the common core of antisocial predisposition, the specific type of criminal behavior is modulated by the configuration of the remaining dimensions, particularly neuroticism and extraversion.

#### 4.2.3. Recidivism and Mediating Mechanisms

Dargis and Koenigs (2018) used the Big Five to identify distinct subgroups of inmates with different profiles of affectivity and behavioral control, associated with varying levels of psychopathology, impulsivity, and substance use. Psychopathy—conceptually correlated with the Big Five through low agreeableness and conscientiousness—is robustly associated with recidivism: the meta-analysis by Gillespie et al. (2023), based on approximately 77,000 participants, reveals significant associations with violent, sexual, and general recidivism, while among adolescents, the behavioral dimensions of psychopathy predict recidivism more strongly than the interpersonal dimensions (Braga et al., 2023).

A relevant example of the integration of these mechanisms in the Romanian context is the study by Rada et al. (2025b), which shows that elevated levels of conscientiousness, agreeableness, and emotional stability are associated with reduced criminogenic risk and less hostile attitudes toward authority—confirming the Big Five profile reported internationally. A distinct finding of the study is the identification of a mediating role of self-efficacy and self-control in the relationship between personality traits and criminogenic behavior, suggesting that intervention targeting cognitive mechanisms may modulate criminal risk independently of the stable personality profile—relevant both for risk assessment and for the design of psychological interventions in the prison setting.

#### 4.2.4. Developmental Continuity

Studies on juvenile delinquency show that these profiles are identifiable early in development: Le Corff and Toupin (2009) found low conscientiousness and agreeableness together with elevated neuroticism among persistent juvenile delinquents, findings confirmed by Van Dam et al. (2005), while the meta-analysis by Tackett et al. (2016) supports the continuity of personality traits from childhood to adulthood. From a developmental perspective, Moffitt (2018) emphasizes the central role of self-control, with early deficits being longitudinally associated with delinquency and difficulties in social adjustment.

#### 4.2.5. Integrative Perspectives and Limitations

In economic crime, the Big Five functions as an indirect explanatory framework, associated with cognitive mechanisms such as unrealistic optimism and confirmation bias (Dearden, 2019), which are correlated with neuroticism and conscientiousness. However, the literature points to methodological limitations related to cross-sectional designs and self-report measures, as well as the constant interaction of personality with social and contextual factors (Farrington et al., 2016). Recent integration of the Big Five with the DSM-5 and HEXACO models has allowed for a more refined explanation through dimensions such as antagonism, dishonesty, and behavioral disinhibition (Sleep et al., 2018; Ashton & Lee, 2020).

**Figure 2:**
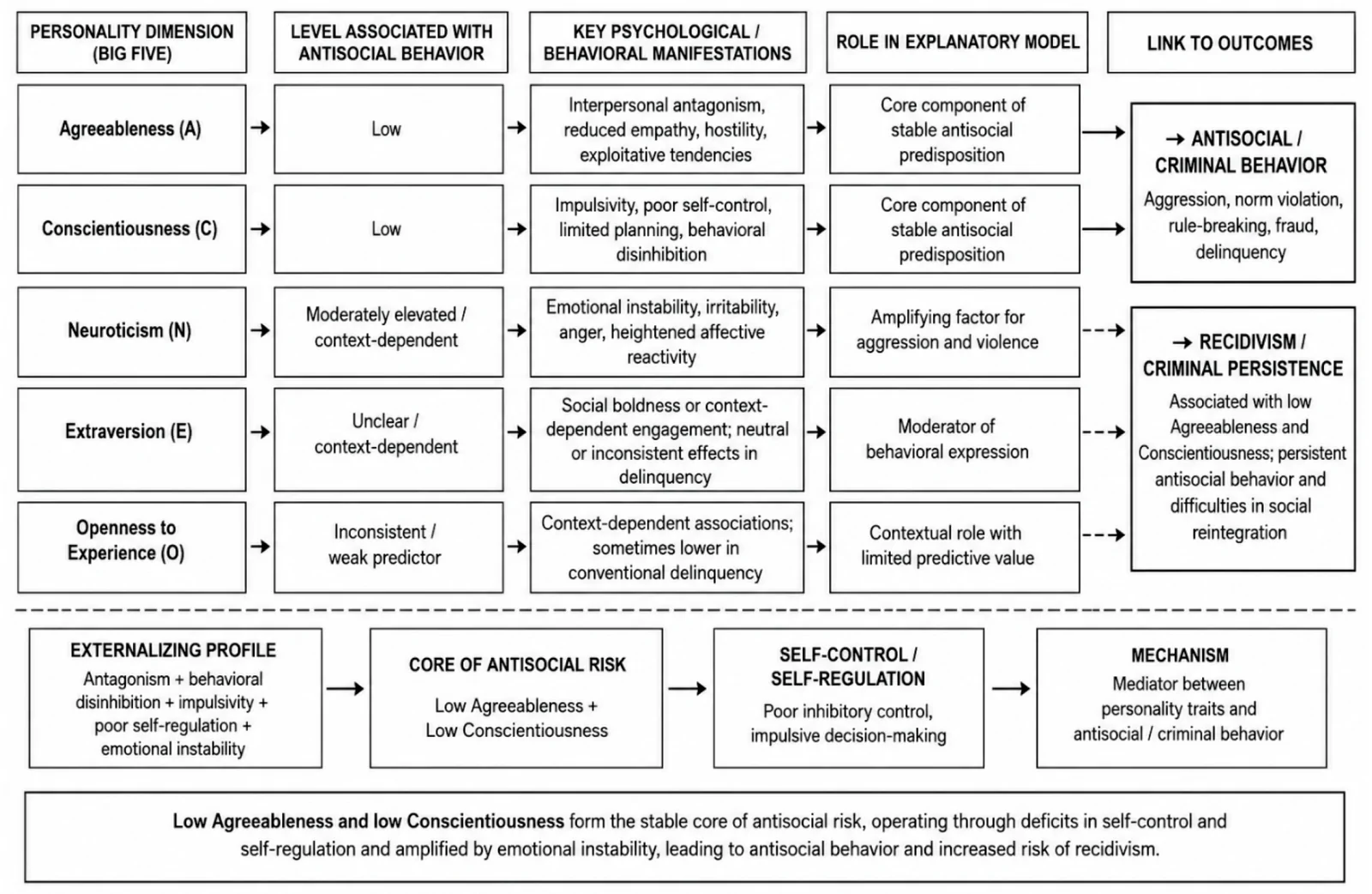
Big Five personality traits, latent mechanisms, and antisocial/criminal behavior outcomes: A conceptual model (Author’s conceptual synthesis).

Figure 2 synthesizes these relationships in a hierarchical explanatory model: low agreeableness and conscientiousness constitute the stable core of antisocial predisposition, which is expressed through an externalizing profile (antagonism, disinhibition, self-regulation deficits) acting as a proximal mechanism. Neuroticism acts as an amplifying factor for emotional reactivity and aggression, extraversion has a context-dependent influence, and openness to experience shows reduced predictive capacity. At the behavioral level, the externalizing profile mediates the relationship between the underlying traits and antisocial manifestations, including persistence and recidivism.

#### 4.2.6. Future Directions

Future research should integrate the Big Five with clinical personality models and maladaptive traits, including HEXACO—particularly useful for economic crime and immoral behaviors—and move from the level of global dimensions to that of facets, to increase predictive precision. Investigating configural models (interactions among traits, e.g., high neuroticism combined with low conscientiousness) and achieving finer differentiation across offense types (violent, sexual, economic, common) would refine existing explanatory and predictive models.

Overall, criminal behavior should be understood as the result of the interaction between personality traits and social, developmental, and situational factors—a premise that supports the use of dimensional models in risk assessment, recidivism prediction, and the design of interventions aimed at self-regulation, self-control, and self-efficacy.

### 4.3. Personality Structures in the HEXACO Model and the Role of Honesty–Humility in Criminal Behavior and Delinquency

The HEXACO model of personality was developed by Ashton and Lee (2007) as an extension of the Big Five model, proposing six fundamental dimensions: Honesty–Humility (H), Emotionality (E), Extraversion (X), Agreeableness (A), Conscientiousness (C), and Openness to Experience (O). A major contribution of the model consists of separating the Honesty–Humility dimension from the Agreeableness factor in the Big Five, allowing for the distinct assessment of tendencies toward sincerity, fairness, and non-exploitation in interpersonal relations—a dimension particularly relevant for explaining antisocial and criminal behaviors (Ashton & Lee, 2007).

#### 4.3.1. Honesty–Humility and Antisocial Behavior

Although the HEXACO model has attracted growing interest in the specialized literature, particularly over the past decade, studies investigating its applicability in the context of criminal behavior remain relatively limited compared with the extensive body of research devoted to the Big Five model; the main relevant empirical contributions identified in the recent literature are presented below.

In the specialized literature, HEXACO is considered one of the most relevant models for understanding antisocial behavior, particularly due to the Honesty–Humility dimension, which is consistently associated with high levels of interpersonal exploitation, manipulation, opportunistic behavior, and violation of social norms (Ashton et al., 2014; Zettler et al., 2020). These relationships have been confirmed both in meta-analytic studies and in empirical research on general and clinical populations.

Studies comparing offenders and non-offenders indicate significant differences across several HEXACO traits. Rolison et al. (2013), in a sample of *N* = 45 male offenders compared with 46 male nonoffenders, show that offenders systematically display lower scores on Honesty–Humility, Conscientiousness, and Extraversion, as well as differences in Agreeableness and Emotionality, a profile associated with impulsivity, interpersonal hostility, and exploitative tendencies. Similarly, Međedović (2017), in a sample of inmates, confirms that criminality is negatively correlated with H, E, A, and C, with the strongest association observed for low Agreeableness. Large-scale confirmation of these associations comes from a recent study of 12,496 Danish participants, in which HEXACO scores were linked to official criminal convictions recorded over a 41-year period, with results confirming robust negative associations between criminality and Honesty–Humility, Emotionality, and Conscientiousness, relatively consistent across offense types (Bader et al., 2025).

Honesty–Humility has been identified as one of the most consistent predictors of antisocial behavior. Low levels of this dimension are associated with a wide range of deviant conduct, including aggression, fraud, deception, and violation of institutional rules, in both social and organizational contexts (Zettler et al., 2020; Ścigała et al., 2022). In organizational settings, low Honesty–Humility (H) and low Conscientiousness (C) are frequently associated with counterproductive behaviors and violations of professional norms (Zappalà et al., 2022).

Regarding Conscientiousness, research indicates that reduced levels are associated with increased impulsivity, poor self-control, and planning difficulties, factors that heighten the risk of disorganized behavior and norm violation (Međedović, 2017). These traits are relevant both for juvenile delinquency and for recidivism.

Studies also show that offenders tend to display lower levels of Extraversion, particularly with regard to sociability and social self-esteem, contrary to older theoretical models (Rolison et al., 2013). This apparent contradiction is explained by differences in content between constructs bearing the same name: whereas Extraversion in Eysenck’s model included a substantial impulsivity component, Extraversion in HEXACO is defined through facets such as sociability, liveliness, social boldness, and social self-esteem—characteristics associated with positive, prosocial engagement in interpersonal settings (Ashton & Lee, 2007; Rolison et al., 2013).

A comparable divergence emerges for Emotionality. Međedović (2017), in a sample of *N* = 256 male convicts, reports a negative correlation between Emotionality and criminality, consistent with low Emotionality (fearlessness, reduced anxiety) as part of the offender profile. Rolison et al. (2013), by contrast, found that incarcerated offenders scored significantly higher than non-offenders specifically on the fearfulness facet of Emotionality (Cohen’s d = −0.53, *p* < 0.05). This is a genuine facet-level contradiction concerning fearfulness and anxiety specifically, not—as an earlier draft of this review mischaracterized it—a contrast between fearlessness and anger or irritability, which are not opposing poles of the same construct. The discrepancy is not resolved by these two studies alone and may reflect differences in offender subpopulations, comparison groups (non-offenders versus normative samples), or unmeasured moderators; it is noted here as an open empirical question.

Regarding Emotionality, low levels are associated with reduced fear and anxiety, which may facilitate risk-taking and antisocial behavior, whereas low Agreeableness reflects tendencies toward hostility and aggressive reactions in conflictual social contexts (Ashton et al., 2014).

In offender populations, these patterns have been further confirmed. Ścigała et al. (2022) show that inmates display significantly lower levels of Honesty–Humility compared with the general population, and that this is associated with aggressive behavior in experimental contexts, although it does not directly predict the type or frequency of offenses.

#### 4.3.2. Differences by Age and Context (Adolescents vs. Adults)

Among adolescents, Honesty–Humility is associated with prosocial behavior and inversely correlated with antisocial behavior in the school environment, with the effect influenced by situational context (Allgaier et al., 2015). Recent research also shows that low levels of this dimension are associated with conduct disorders and externalizing behaviors in adolescents, suggesting its role as a risk factor for the development of delinquency (Muris et al., 2022, 2023).

Regarding delinquency in adults, HEXACO has demonstrated incremental validity over other models. Dunlop et al. (2012) show that both Honesty–Humility and dimensions from Eysenck’s model (e.g., psychoticism) contribute significantly to the prediction of delinquent behavior. Facet-level analysis provides a finer and more precise perspective: Petrovic et al. (2014) show that not only the broad domains but also specific HEXACO facets—particularly those related to sincerity, fairness, and avoidance of exploitation—contribute distinctly to the prediction of criminal behavior, a pattern also confirmed in the context of occupational offenses, where personality traits interact with situational factors such as opportunity and risk (Van Gelder & de Vries, 2016). The mechanism through which Honesty–Humility reduces involvement in criminal behavior was investigated experimentally by Chatzimike Levidi et al. (2024), who showed that higher levels of this dimension increase perceived risk of apprehension and anticipated negative affect, both of which in turn reduce the intention to commit an offense.

#### 4.3.3. Limitations of the Model and Complementary Perspectives

Overall, the HEXACO model offers a detailed perspective on the stable personality predispositions involved in criminal behavior, allowing for differentiation among types of delinquency (violent, economic, or non-violent) and the identification of distinct psychological profiles, defined by a common core of low levels of Honesty–Humility, Conscientiousness, and Agreeableness—a core complemented, depending on the type of deviant behavior, by variable levels of Emotionality and Extraversion, as illustrated in Table 3.

The main limitation of the model, however, lies in the fact that it predominantly describes the relatively stable structure of personality without sufficiently capturing the situational variation in behavior. In this respect, the DIAMONDS taxonomy (Rauthmann et al., 2014) offers a complementary framework, describing the essential situational dimensions in which personality traits are activated and influence behavior, contributing to a more complete understanding of the person–situation interaction in explaining delinquency.

### 4.4. Dark Traits, Psychopathy, and the Structure of Antisocial Personality

#### 4.4.1. Conceptual Framework and Explanatory Models

The Dark Triad of personality was introduced by Paulhus and Williams (2002), encompassing Machiavellianism, narcissism, and subclinical psychopathy. These traits are conceptualized as distinct, yet intercorrelated dimensions reflecting antisocial tendencies characterized by self-interest, lack of empathy, and instrumental use of interpersonal relationships.

Machiavellianism is defined by strategic manipulation, interpersonal cynicism, and orientation toward personal goals achieved through exploitative means (D. N. Jones & Paulhus, 2014). Narcissism involves grandiosity, a need for admiration, and sensitivity to status, whereas subclinical psychopathy is associated with impulsivity, reduced affectivity, and persistent antisocial behavior.

Furnham et al. (2013) argue for a contextual interpretation of the effects of Dark Triad traits, showing that the three traits display differentiated correlates depending on the context in which the three traits are measured and the behavioral criteria examined. Although the Dark Triad dimensions are intercorrelated and share a common core of manipulation devoid of empathy, their effects are not uniform: each trait predicts distinct types of antisocial and deviant behavior, and the magnitude of these associations varies by domain—interpersonal relationships, occupational performance, academic conduct, or criminality. The authors also note that Dark Triad traits are more prevalent among men than women, are predominantly associated with the Agreeableness factor in the Big Five model and with the Honesty–Humility factor in HEXACO, and are generally linked to a wide range of negative psychosocial consequences.

The “Dark Core of Personality” model proposed by Moshagen et al. (2018) introduces the concept of a general dark factor of personality (D-factor), which accounts for the overlap among dark traits through a common core defined as the tendency to maximize one’s own utility, irrespective of—or even at the expense of—others, accompanied by belief systems that serve as justifications for such behavior. From a criminological perspective, the relevance of the D-factor lies in its empirically demonstrated predictive capacity: D shows significant correlations with aggression and with criminal and delinquent behavior and explains incremental variance in antisocial behavioral criteria even after controlling for Big Five dimensions. Moreover, validation studies indicate that D exhibits high temporal stability and that the prediction of specific antisocial behaviors is better explained by this general factor than by individual dark traits taken separately, granting it theoretical and applied value superior to models that treat Machiavellianism, narcissism, and psychopathy as independent predictors.

#### 4.4.2. The Dark Triad and Criminal Behavior

Studies indicate consistent differentiations among Dark Triad traits in relation to types of deviant behavior: Machiavellianism is predominantly associated with instrumental criminality and manipulation/deception-type deviance, narcissism with reactive aggression in response to ego threat, and psychopathy with impulsive-antisocial and violent behaviors, being the most robust predictor of violence among the three traits (Muris et al., 2017; Blais et al., 2014; Hare & Neumann, 2019). Meta-analyses confirm a consistent association between Dark Triad traits and criminal behavior, including aggression, fraud, norm violation, and recidivism (Zhu & Jin, 2021).

Wright et al. (2017) investigate the relationship between the Dark Triad and low self-control in explaining criminal behavior, showing that the two independently predict delinquent behavior, with psychopathy identified as the strongest predictor of violent delinquency and severe antisocial behavior; the Dark Triad explains incremental variance in criminal behavior even after controlling for self-control, suggesting independent contributions of personality beyond impulsivity, with more pronounced effects in interpersonal and violent offenses, whereas self-control has a more general role.

Alsheikh Ali (2020), in a sample of adolescents, demonstrated that psychopathy is the strongest predictor of delinquency, followed by Machiavellianism, while narcissism shows a smaller effect. The study also shows that demographic variables contribute significantly less to the prediction of delinquency compared with personality traits, reinforcing the view that individual psychological factors play a primary explanatory role relative to sociodemographic characteristics in understanding juvenile delinquent behavior.

Hampejs et al. (2025) conducted a preregistered, systematic meta-analysis focused exclusively on the association between the Dark Triad and criminal behavior resulting in arrest. The three-level analysis, based on 302 effect sizes drawn from 39 studies across 15 countries (*N =* 15,862), confirmed a significant positive association between Dark Triad traits and criminal behavior, with psychopathy as the strongest predictor, followed by Machiavellianism and narcissism. Participant age was identified as a significant moderator of effect heterogeneity across studies, and the authors emphasize the need for ecologically valid samples—composed of offenders rather than students—to obtain more precise estimates of the associations between dark traits and criminality.

#### 4.4.3. Extending the Model: The Dark Tetrad

The Dark Tetrad (Machiavellianism, narcissism, psychopathy, and sadism) extends the explanatory framework of antisocial behavior. Međedović (2024) shows that all Dark Tetrad traits are associated with antisocial behavior, but Machiavellianism is the strongest predictor of recidivism and persistent criminal conduct, through mechanisms of manipulation and strategic planning.

Hurezan et al. (2024) simultaneously examine dark traits and “bright” traits (adaptive Big Five). The results show that dark dimensions are positively associated with criminal behavior, whereas conscientiousness and emotional stability play a protective role, with the integrated model showing superior predictive capacity compared to separate models.

A recent study conducted on a Romanian population (Rada & Forțu, 2026) examined the relationship among Dark Triad traits, emotional stability, and aggression in a sample of 250 male inmates convicted of violent offenses. The results revealed a clear functional differentiation of traits: psychopathy significantly predicted physical aggression and anger, explaining 44.4% of the variance in physical aggression, while Machiavellianism was the strongest predictor of verbal aggression and hostility; narcissism showed no significant contribution to physical aggression. Emotional stability was negatively correlated with all dimensions of aggression, suggesting a protective role of emotional regulation. In addition, higher levels of physical aggression were associated with a lower likelihood of participation in educational and vocational training activities within the institution, underscoring the practical implications of these traits for the social reintegration of violent inmates.

#### 4.4.4. Psychopathy as an Explanatory Core

Psychopathy is defined as a stable pattern of interpersonal, affective, and behavioral traits: superficial charm, manipulation, lack of empathy, impulsivity, and persistent violation of social norms (Patrick et al., 2009). Brugués and Caparrós (2021) show that antisocial and aggressive-sadistic traits are associated with moral disengagement mechanisms, such as moral justification, advantageous comparison, and dehumanization, mechanisms that contribute to violent behavior and recidivism.

The meta-analytic literature provides consistent evidence for the role of psychopathy as a stable risk factor across multiple forms of criminal behavior. Regarding type of violence, Blais et al. (2014) demonstrated that psychopathy shows moderate and significant associations with both instrumental and reactive violence, with differentiation at the facet level: the interpersonal dimension is more relevant to instrumental violence, whereas the social deviance factor is more important for reactive violence. The relevance of psychopathy extends to juvenile populations as well: Geerlings et al. (2020) confirmed, through a three-level meta-analysis, a moderate association between psychopathic traits and juvenile delinquency, with stronger effects for impulsivity than for callous-unemotional traits. At the facet level of the PCL:YV, Braga et al. (2023) showed that the lifestyle and antisocial facets are the most consistent predictors of violent and general recidivism among young offenders, varying according to the follow-up interval. Regarding sexual offenses, Moretti et al. (2024) confirmed, through a systematic review based on 14 studies, that psychopathic traits assessed via the PCL-R are associated with an increased risk of general recidivism among adult male sexual offenders, although the association with sexual recidivism specifically remains less conclusive. Hare and Neumann (2019) synthesize this empirical base, confirming that psychopathy remains one of the most robust predictors of violence and recidivism in the contemporary criminological literature.

Because psychopathy is treated throughout this review as a dimensional personality construct, its developmental trajectory across time is directly relevant to the stable-traits-versus-dynamic-mechanisms distinction proposed in the integrative model. Classic developmental research on personality more broadly indicates that trait rank-order consistency increases progressively from childhood into mid-adulthood, stabilizing rather than remaining fixed from an early age (Roberts & DelVecchio, 2000). Within offender populations specifically, longitudinal evidence directly addressing psychopathic-trait development remains considerably scarcer than cross-sectional research, but the few available studies converge on a pattern of partial, not absolute, stability. Using the seven-year Pathways to Desistance sample of adjudicated adolescents, Lasko and Chester (2020) found that higher initial psychopathy scores predicted steeper subsequent increases in inhibitory control and anger regulation over time, particularly among offenders who went on to reoffend less—a compensatory developmental pattern consistent with the broader distinction, central to the present model, between relatively stable dispositional traits and modifiable self-regulatory mechanisms. Similarly, in a shorter-term study of adolescents in youth welfare and juvenile justice institutions, Hachtel et al. (2022) reported that self-reported psychopathic traits showed only moderate stability over an approximately one-year interval, with meaningful individual-level change rather than fixed trait expression. Taken together, these findings suggest that even the most putatively stable maladaptive configuration examined in this review is subject to measurable developmental change in offender populations—a pattern that reinforces, rather than undermines, the rationale for targeting dynamic mechanisms in intervention, and one that longitudinal research on the other frameworks reviewed here (FPI, Big Five, HEXACO, criminogenic cognitions) has yet to establish with comparable specificity.

Bazinet et al. (2022) retrospectively investigated the contribution of psychopathic traits and substance use to the prediction of recidivism among high-risk sexual offenders, in a sample of 579 inmates enrolled in specialized treatment programs in Canada. Alcohol and drug use were identified as factors amplifying the likelihood of recidivism, and self-reported history of drug-related problems proved to be a significant predictor of recidivism among sexual offenders. The authors note that psychopathy remains the primary explanatory core of recidivism, but that substance use increases the predictive accuracy of the model, underscoring the importance of integrating dynamic criminogenic factors alongside stable personality traits in risk assessment for this category of offenders.

#### 4.4.5. Integration of Dimensional Models

Gaughan et al. (2012) comparatively examined the utility of the HEXACO-PI-R and NEO PI-R in assessing psychopathy in a sample of 290 students, finding that both inventories explain substantial proportions of variance in psychopathy scores, with a modest advantage for the HEXACO-PI-R. The authors specifically discuss the role of the Emotionality dimension in HEXACO, which functions differently from Neuroticism in the NEO PI-R in relation to psychopathic traits, suggesting that the HEXACO model captures additional nuances relevant to understanding psychopathy as an extreme variant of normal personality dimensions.

Lee and Ashton (2014) show that the Dark Triad is strongly associated with the HEXACO model, particularly through the Honesty–Humility factor, which represents the inverse core of dark traits. This integration explains the mechanisms of criminal behavior through interpersonal exploitation, moral deficit, and reduced behavioral inhibition.

**Figure 3:**
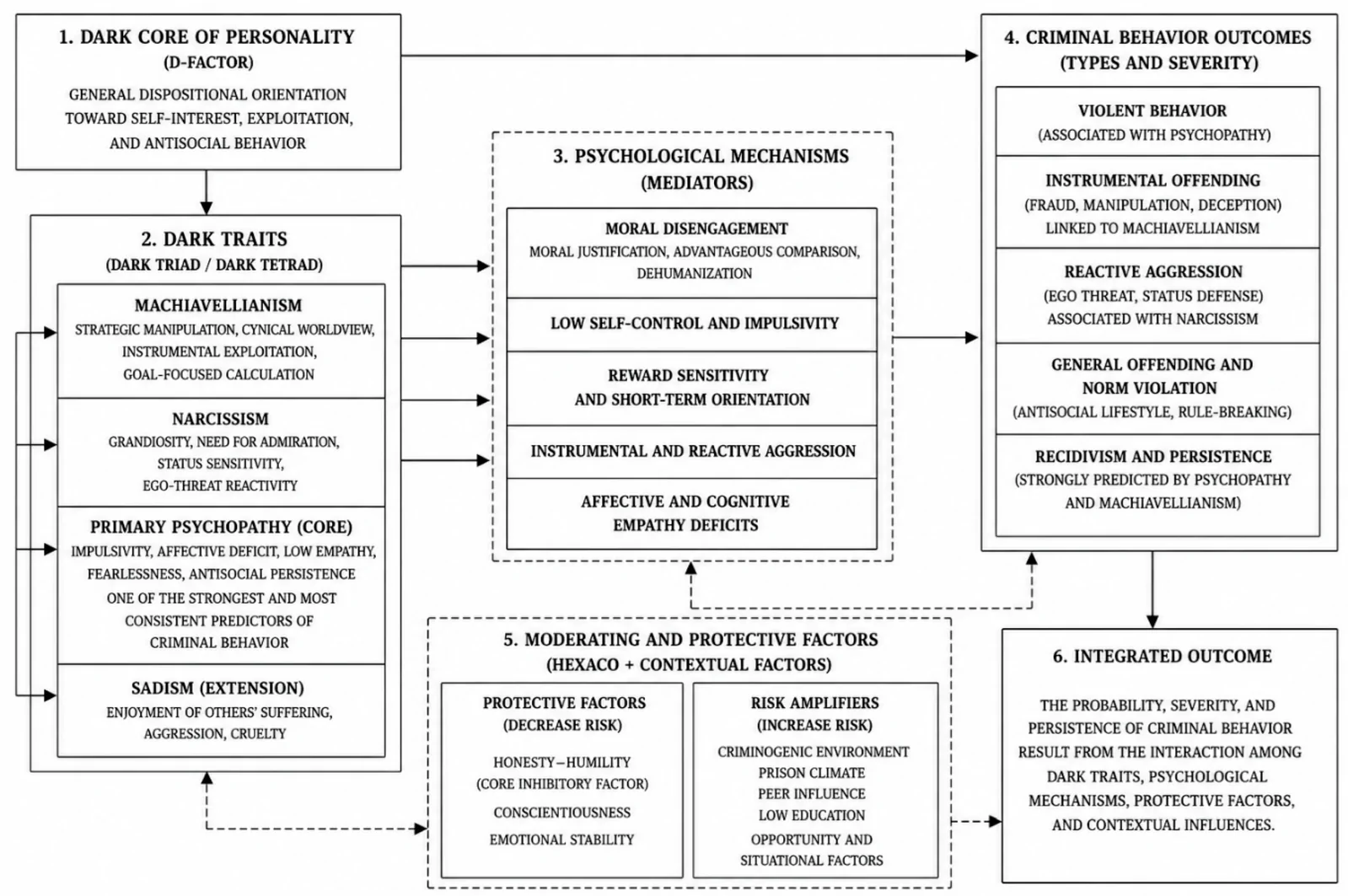
Integrative model of dark traits and criminal behavior. Solid arrows indicate direct predictive relationships between the model’s variables, while dashed arrows represent indirect effects, including mediating mechanisms and moderating influences. Dashed-outline boxes delineate clusters of psychological mechanisms and contextual factors that function as mediators or moderators in the relationship between dark personality traits and criminal behavior.

In conclusion, the literature converges on a model in which psychopathy represents the central explanatory core of criminal behavior, while Machiavellianism, narcissism, and contextual factors (self-control, substance use, Big Five traits) contribute differentially depending on the type of antisocial behavior and the situational context, as illustrated in the figure below (Figure 3).

### 4.5. Criminogenic Cognitions and Cognitive Mechanisms Associated with Criminal Behavior

#### 4.5.1. Defining the Concept and Its Criminological Relevance

Criminogenic cognitions represent a central construct in explaining persistent criminal behavior, defined as dysfunctional thinking patterns that facilitate, justify, or legitimize the violation of social norms. These cognitive structures influence the decision-making process, the interpretation of social situations, and the selection of behavioral responses, contributing to the maintenance of antisocial behavior and to recidivism (Tafrate & Mitchell, 2022; Dina et al., 2022).

In the contemporary literature, criminogenic cognitions are included among the main dynamic factors of the Risk-Need-Responsivity (RNR) model, being regarded as priority targets of correctional interventions (Bonta & Andrews, 2017). Unlike stable personality traits, criminogenic cognitions represent dynamic constructs, modifiable through psychological intervention, which gives them particular practical relevance in the design of cognitive-behavioral programs aimed at reducing recidivism and developing behavioral self-regulation (Taxman et al., 2014).

#### 4.5.2. Assessing Criminogenic Cognitions: The Criminogenic Cognitions Scale (CCS)

Among the instruments used to assess criminogenic cognitions, one is the Criminogenic Cognitions Scale (CCS), developed by Tangney and colleagues within research on moral emotions and antisocial behavior (Tangney et al., 2007, 2012). The scale contains 25 items rated on a four-point Likert scale and measures five dimensions of criminogenic thinking: a sense of entitlement, failure to accept responsibility, short-term orientation, insensitivity to the impact of crime on victims, and negative attitudes toward authority. Initial studies demonstrated satisfactory psychometric properties—adequate internal consistency, construct validity, and predictive utility for antisocial behavior and self-regulation difficulties (Tangney et al., 2012).

The CCS was selected as the primary focus of this section given its brevity, its explicit theoretical grounding in entitlement and responsibility-denial constructs, and its recent validation in the Romanian incarcerated population discussed in the General Discussion (Rada et al., 2026). Other established instruments assessing related but non-identical criminal-thinking constructs were consulted for context: the Psychological Inventory of Criminal Thinking Styles (PICTS), discussed above in relation to interpersonal hostility, and the Criminal Sentiments Scale—Modified (CSS-M; Shields & Simourd, 1991; Simourd, 1997), which assesses attitudes toward law, courts, and police alongside identification with criminal others and tolerance for law violation. A systematic comparison of the convergent and discriminant validity of these instruments was beyond the scope of the present narrative synthesis.

The psychometric properties of the CCS have since been confirmed in different cultural contexts: Jamil and Fatima (2018) translated and validated the scale into Urdu in a sample of 452 adolescents, while Shahrakht et al. (2019) confirmed the factorial structure and reliability of the instrument in a sample of 397 Iranian university students. In the European prison context, Pereira et al. (2025) evaluated the CCS in a sample of 364 Portuguese inmates, identifying a stable factorial structure and significant associations between criminogenic cognitions, reduced self-control, and moral disengagement.

In the Romanian context, the first validation of the CCS, in a sample of 460 individuals deprived of liberty, was carried out by Rada et al. (2026). The results indicated moderate validity of the instrument and showed that educational level, marital status, type of offense, and drug use significantly influenced the level of criminogenic cognitions, with the highest scores recorded among individuals convicted of sexual offenses and those who perceived their sentence as unjust—characteristics associated in the literature with an increased risk of recidivism. These findings suggest that the profile of criminogenic cognitions varies according to specific sociodemographic and judicial characteristics, with implications for the differentiation of correctional interventions.

#### 4.5.3. Criminogenic Cognitions and Moral Disengagement

Shulman et al. (2011), in a longitudinal study of 1169 serious juvenile offenders, demonstrated that reduced moral disengagement is associated with a decrease in antisocial behavior, whereas high levels predict the persistence of criminal conduct. Similar findings were reported by Van der Velden et al. (2010), who showed that the way adolescents interpret moral situations and justify certain actions significantly influences their social behavior, with deficits in moral reasoning and the tendency to use cognitive justifications being associated with higher levels of antisocial conduct. More recently, Chen and Sutton (2025) demonstrated the existence of a reciprocal relationship between moral disengagement and antisocial behavior, with the two processes reinforcing each other over the course of development.

The moral-disengagement processes discussed above plausibly rest on an underlying neurodevelopmental substrate, illustrated here through two frequently cited sources rather than through an exhaustive review of the neurocriminology literature, which lies outside the scope of the present synthesis. Blair (2013) examines the neurobiological basis of psychopathic traits in children and adolescents, showing that functional deficits in the amygdala and the ventromedial prefrontal cortex impair the recognition of others’ distress, the development of empathy, and learning from the negative consequences of one’s own actions, contributing to callous-unemotional traits, aggression, and persistent antisocial behavior; the author argues for a neurodevelopmental component of psychopathy and the need for early intervention. In the same vein, Glenn and Raine (2014), through a review of evidence from neurocriminology, show that structural and functional abnormalities in brain regions involved in impulse control, emotion processing, empathy, and decision-making are associated with aggression and antisocial behavior, with neurobiological predispositions interacting with environmental factors—carrying implications for risk assessment, violence prevention, and the development of interventions aimed at reducing recidivism.

#### 4.5.4. Criminogenic Cognitions, Personality, and Interpersonal Hostility

Numerous studies highlight the interplay between criminogenic cognitions and personality traits. Hurezan et al. (2024) show that dark personality dimensions, associated with self-regulation deficits, contribute to the development and maintenance of criminal behavior and recidivism, while Brugués and Caparrós (2021) show that Dark Triad traits and moral disengagement mechanisms are associated with the risk of violence and recidivism among incarcerated individuals. Hauser et al. (2023) concluded that offenders with high levels of psychopathy show significant difficulties in processing social and moral information, which favors the persistence of antisocial behavior.

Carr et al. (2019) examined criminal thinking as a risk factor for aggression among psychiatric inpatients, using the Psychological Inventory of Criminal Thinking Styles (PICTS): the Denial of Harm scale—an indicator of neutralization and hostility—significantly predicted the rate of aggressive incidents, while the BPRS Hostile Suspiciousness scale was a significant predictor of both aggression and violence, confirming the role of interpersonal hostility as a risk factor. Further empirical support comes from the study by Rada et al. (2025a), in a sample of 857 Romanian inmates, who identified a psychological profile characterized by negative emotionality, anxiety, aggression, and reduced self-efficacy—factors that favor hostile interpretations of the environment, externalization of responsibility, and the consolidation of criminogenic cognitions.

#### 4.5.5. Criminogenic Cognitions and Executive Functions

Greenfield and Valliant (2007) compared 20 violent offenders, 19 non-violent offenders, and 20 individuals with no criminal record, using the Porteus Maze Test (executive functions), the Defining Issues Test (moral reasoning), and the MMPI (personality). Violent offenders differed by showing weaker performance on the Porteus Maze Test and higher scores on the Psychopathic Deviate (Pd) scale of the MMPI, with the discriminant model correctly classifying 97% of participants; although violent offenders displayed a relatively mature level of moral reasoning, violent offenders showed more pronounced oppositional attitudes toward authority, as well as higher levels of depression, psychopathic deviation, and social introversion compared with the control group. The results suggest that violent criminality is associated mainly with particularities of executive functioning, personality, and social attitudes, rather than with general deficiencies in moral reasoning.

Seruca and Silva (2015) demonstrated that recidivists show significant deficits in mental flexibility compared with individuals with no criminal record. In a subsequent study, the same authors Seruca and Silva (2016) assessed executive functions in 42 offenders—violent, property, and trafficking offenders—and 28 non-offenders, finding significantly weaker performance on measures of planning among violent offenders, as well as significant correlations between anger control, mental flexibility, and interference inhibition within this subgroup, suggesting that executive control deficits—particularly in cognitive flexibility and inhibition—contribute to impaired anger regulation and the manifestation of violent behavior.

#### 4.5.6. Criminogenic Cognitions and Recidivism

The specialized literature indicates that criminal thinking represents one of the most important dynamic predictors of recidivism. Bourke et al. (2013) demonstrated that the relationship between criminal cognitions and non-violent recidivism is moderated by personality traits, with criminal thinking not being an independent predictor: using Eysenck’s PEN model, the authors showed that high psychoticism, in interaction with elevated criminal cognitions, increased the risk of recidivism, while high extraversion and neuroticism were associated with a reduced risk—findings that indicate the need to assess the interaction between cognitions and personality traits, rather than their independent effects alone.

In a separate study focused on violent recidivism, Boduszek et al. (2013) showed that extraversion, interpersonal hostility, and the cognitive centrality of criminal identity contribute significantly to differentiating violent from non-violent offenders, suggesting that the psychological profile of violent offenders involves not only dysfunctional thinking patterns but also a cognitively consolidated criminal identity.

Walters (2022), in a meta-analysis of the developmental origins of criminal thinking in adults, shows that early adverse experiences, inadequate parenting practices, self-control difficulties, and social adjustment problems contribute to the formation of criminal thinking patterns, which are not an isolated phenomenon but the outcome of a developmental process shaped by individual and social factors—the author recommending preventive interventions aimed at social competencies and self-regulation. More recently, Walters (2023) showed that the interaction between proactive criminal thinking and time spent in prison predicts a greater number of institutional infractions, which in turn increase the likelihood of recidivism after release.

Criminogenic thinking, however, is not specific exclusively to incarcerated populations: Mandracchia and Gulledge (2024) demonstrated that high levels of criminogenic thinking are associated with employment status and dysfunctional workplace behaviors among law enforcement officers. Although this population is not criminal in the traditional sense, the finding carries important theoretical relevance: the fact that criminogenic thinking patterns also appear in non-offender populations with specific occupational risk suggests that criminogenic thinking patterns reflect broader psychological characteristics related to cognitive-behavioral regulation and self-regulation, rather than merely being a product of criminal experience or incarceration, thereby supporting the predictive value of the construct beyond the prison context.

#### 4.5.7. Implications for Assessing the Risk of Violent Recidivism

The body of empirical evidence indicates that criminogenic cognitions represent an essential dynamic risk factor for the assessment and management of recidivism. Criminogenic cognitions are associated with moral disengagement, interpersonal hostility, dark personality traits, and executive function deficits, all of which are variables implicated in the maintenance of violent behavior and recidivism. Because they are susceptible to modification through cognitive-behavioral interventions, criminogenic cognitions constitute a priority target of correctional programs aimed at reducing recidivism risk and facilitating social reintegration (Landenberger & Lipsey, 2005).

**Figure 4:**
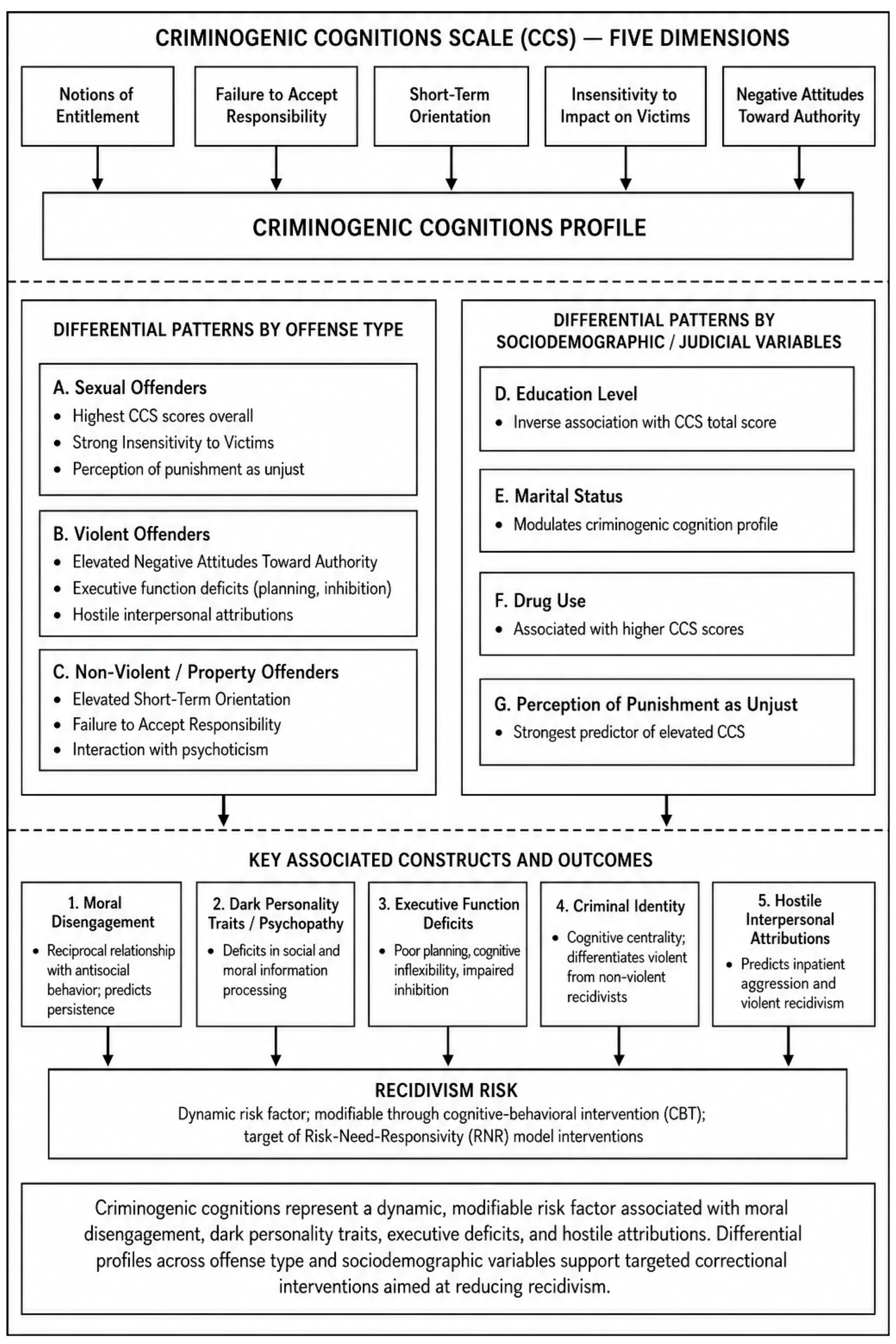
Conceptual Framework of Criminogenic Cognitions and Their Association with Recidivism Risk.

A synthesis of the conceptualization of criminogenic cognitions and their association with recidivism risk is presented in Figure 4.

## 5. General Discussion

The present narrative integrative review analyzed the literature on personality and criminogenic cognitions in incarcerated populations through five conceptual frameworks—the Freiburg Personality Inventory (FPI), the Big Five model, the HEXACO model, the Dark Triad/Dark Tetrad constructs and psychopathy, and the theory of criminogenic cognitions—organized around the integrative conceptual model proposed in Section 3. In what follows, the findings are discussed in relation to the three levels of this model, to the convergent heterogeneity of the incarcerated population identified across all five frameworks, to the conceptual convergences among models, and, finally, to common methodological limitations and future research directions.

### 5.1. From Stable Traits to Dynamic Mechanisms: A Synthesis Across the Three Levels

At the dispositional level of personality, the literature examined through the FPI, Big Five, and HEXACO converges on a relatively consistent core of antisocial vulnerability: elevated aggression and emotional excitability, low conscientiousness and agreeableness, and reduced levels of Honesty–Humility (Hesener & Hilse, 1983; Hosser et al., 2008; Miller & Lynam, 2001; Ashton & Lee, 2007). These traits do not, however, appear sufficient in themselves to explain the variability of criminal behavior, a point suggested by studies indicating the role of intervening dynamic mechanisms. Thus, in a study of 857 Romanian inmates assessed with the FPI, self-efficacy was identified as a mediator of the relationship between personality traits and the coping strategies used in adapting to detention (Rada et al., 2025a), and a similar mediating role of self-efficacy and self-control was confirmed, in the same population, for the relationship between Big Five traits and criminogenic behavior (Rada et al., 2025b). These findings are consistent with those of Chatzimike Levidi et al. (2024), who show experimentally that Honesty–Humility reduces criminal intent by increasing perceived risk of apprehension and anticipated negative affect—a dynamic mechanism similar to that identified for self-efficacy and coping—as well as with those of Wright et al. (2017), who show that Dark Triad traits and low self-control independently predict delinquent behavior, and with those of Bourke et al. (2013), who demonstrate that the relationship between criminal cognitions and non-violent recidivism is moderated by personality traits. Taken together, these findings—each testing a distinct relationship (personality–coping mediation, personality–criminogenic behavior mediation, an experimental effect on criminal intent, independent parallel prediction of delinquency, and moderation of the cognition–recidivism link, respectively)—converge thematically with the distinction, well established within the Risk-Need-Responsivity model, between static and dynamic risk factors that are modifiable through intervention (Bonta & Andrews, 2017). This distinction is consistent with, rather than a direct demonstration of, the idea central to the conceptual model proposed in this work: that the dispositional level may exert much of its influence on behavior indirectly, through self-regulatory mechanisms, rather than in a simple, direct fashion.

At the level of maladaptive personality configurations, the literature indicates a functional differentiation of dark traits in relation to the type of aggressive behavior: psychopathy is predominantly associated with instrumental and impulsive-antisocial violence (Blais et al., 2014; Muris et al., 2017), whereas Machiavellianism is more strongly linked to manipulation- and deception-type deviance (Hampejs et al., 2025; Međedović, 2024). This differentiation is further illustrated by a study of 250 Romanian inmates convicted of violent offenses, in which psychopathy significantly predicted physical aggression (detailed in Section 4.4), while Machiavellianism was the strongest predictor of verbal aggression and hostility, with emotional stability acting protectively against all dimensions of aggression assessed (Rada & Forțu, 2026). Similar findings, regarding the distinct role of the behavioral versus interpersonal facets of psychopathy in predicting recidivism, have been reported by Braga et al. (2023), reinforcing the notion that the level of maladaptive configurations cannot be treated as a unitary block but requires differentiated analysis by trait type and by the type of resulting behavior.

At the level of proximal cognitive mechanisms, cross-cultural validations of the Criminogenic Cognitions Scale—conducted on samples from Pakistan (Jamil & Fatima, 2018), Iran (Shahrakht et al., 2019), and Portugal (Pereira et al., 2025)—confirm a relatively stable factorial structure of the construct across cultural boundaries. In the Romanian context, the first validation of the scale, in individuals deprived of liberty, showed significant variation in criminogenic cognitions according to participants’ sociodemographic and judicial characteristics (Rada et al., 2026)—findings compatible with those reported by Walters (2022, 2023) regarding the role of individual and institutional factors in the formation and persistence of criminal thinking.

Taken together, these findings—drawn from different populations, instruments, cultural contexts, and analytic designs—provide convergent, though not uniform, support for the three-level conceptual model proposed in Section 3, suggesting that dispositional traits, maladaptive configurations, and criminogenic cognitions do not operate in isolation. Rather than establishing a single validated mediation pathway to criminal behavior, the evidence reviewed here is consistent with the possibility that self-efficacy and self-control may represent proximal mechanisms through which dispositional vulnerabilities are translated into maladaptive coping, criminogenic cognitions, and, in some contexts, criminal behavior.

### 5.2. Convergent Heterogeneity of the Incarcerated Population

A second cross-cutting finding, observed across all five conceptual frameworks analyzed, is the psychological heterogeneity of the incarcerated population. At the level of the FPI, cluster analyses by Ille et al. (2005) identified three distinct profiles—ranging from pronounced aggression and emotional instability to scores close to those of the general population. At the level of the Big Five, Dargis and Koenigs (2018) confirmed, through a similar approach, the existence of distinct inmate subgroups with different profiles of affectivity and behavioral control. The HEXACO model adds a further, situational type of differentiation: the offense profile varies systematically according to the type of deviant behavior (fraud, violence, juvenile delinquency, impulsive offenses), with Honesty–Humility and Conscientiousness remaining low across all cases, while Emotionality and Extraversion vary considerably. The functional differentiation of Dark Triad traits according to type of aggression (Rada & Forțu, 2026) and the variation in criminogenic cognitions according to offense type and sociodemographic characteristics (Rada et al., 2026) confirm, within the Romanian prison population, the same pattern of heterogeneity observed internationally.

The convergence of these findings—obtained through different instruments, populations, and methodologies—supports the conclusion that the incarcerated population cannot be reduced to a single offender personality profile, but must instead be understood through a dimensional and typological perspective, in which common dispositional vulnerabilities (aggression, self-regulation deficits, low honesty–humility) combine differently depending on the type of criminal behavior, the institutional context, and individual characteristics.

### 5.3. Conceptual Convergences Among the Theoretical Frameworks Analyzed

Beyond the empirical convergence regarding heterogeneity, the literature analyzed also reveals direct conceptual convergences among the five theoretical frameworks, suggesting that the five frameworks do not describe independent phenomena but rather partially overlapping facets of a multilevel architecture of antisocial vulnerability. The association between the Dark Triad and the Honesty–Humility factor in HEXACO illustrates this pattern (Lee & Ashton, 2014).

Furnham et al. (2013) similarly confirm that Dark Triad traits are predominantly associated with the Agreeableness factor in the Big Five. The “Dark Core of Personality” model by Moshagen et al. (2018) systematizes these overlaps through a general factor (D) that explains incremental variance in antisocial behavior even after controlling for Big Five dimensions, suggesting the existence of a common core that transcends the boundaries between models.

These conceptual convergences provide additional support for the integrative model proposed in this work: rather than competing explanatory frameworks, the FPI, Big Five, HEXACO, Dark Triad/Tetrad, and criminogenic cognitions appear to describe, from different angles and at different degrees of proximity to manifest behavior, complementary levels of a multilevel architecture of antisocial vulnerability, rather than interchangeable indicators of a single underlying construct.

### 5.4. Practical Implications

The three-level model proposed here carries direct implications for correctional assessment and intervention practice. First, because stable dispositional traits (Conceptual Level I) show only indirect, mediated associations with criminal behavior, psychological profiling based exclusively on broad-band trait measures (FPI, Big Five, HEXACO) is unlikely, by itself, to yield actionable intervention targets; assessment protocols should instead incorporate measures of the dynamic mechanisms identified here as mediators—self-efficacy, self-control, and coping—consistent with the dynamic risk factors emphasized by the Risk-Need-Responsivity model (Bonta & Andrews, 2017). Second, the functional differentiation observed at the level of maladaptive configurations (Conceptual Level II)—psychopathy predicting predominantly instrumental and impulsive-antisocial violence, Machiavellianism predicting predominantly manipulation- and deception-type deviance—suggests that risk-assessment protocols and intervention planning should be tailored to the specific maladaptive configuration identified in each individual, rather than treating “dark personality” as an undifferentiated risk marker. Third, given the central mediating role attributed here to criminogenic cognitions (Conceptual Level III) and the availability of a validated instrument in the Romanian context (Rada et al., 2026), cognitive-restructuring interventions targeting criminal thinking styles and neutralization mechanisms represent a directly actionable target, potentially more modifiable in the short term than interventions aimed at the underlying personality traits themselves. These implications should nonetheless be regarded as hypotheses derived from the conceptual model proposed in this review, pending the direct empirical validation discussed below.

### 5.5. Common Methodological Limitations and Future Directions

The integrated analysis of the five theoretical frameworks also reveals methodological limitations common to the literature as a whole. First, most of the instruments discussed rely on self-report, making them vulnerable to social desirability and to the influence of the institutional context on responses—an effect explicitly documented for the FPI by Hesener and Hilse (1983) and Kury (1983), but plausibly generalizable to all self-assessment instruments used in the prison environment. Second, research remains geographically unevenly distributed: the FPI is used almost exclusively in German-speaking countries, and cross-cultural validations of the CCS come predominantly from Asia and Western Europe, with populations from Eastern Europe being far less represented—a gap that the recent series of studies conducted on the Romanian prison population (Rada et al., 2025a, 2025b, 2026; Rada & Forțu, 2026) is beginning to address, without eliminating the need for larger samples from other countries in the region.

Third, predominantly cross-sectional designs limit the ability to establish the direction of the relationships among personality traits, criminogenic cognitions, and criminal behavior; the longitudinal studies available (Shulman et al., 2011; Tackett et al., 2016; Chen & Sutton, 2025; Walters, 2023) remain relatively few compared with the volume of cross-sectional research. This scarcity is not uniform across the five frameworks, however: as detailed in Section 4.4, longitudinal evidence on the developmental trajectory of psychopathic traits specifically—though still limited—does exist and indicates partial rather than absolute stability (Roberts & DelVecchio, 2000; Lasko & Chester, 2020; Hachtel et al., 2022). No comparable body of longitudinal trait-development research was identified for the FPI, Big Five, or HEXACO frameworks in offender populations specifically, which remains a genuine gap in the literature rather than one this review can resolve. Finally, although the conceptual model proposed in this work is supported by empirical convergences drawn from independent sources, its direct verification through a single integrated design—assessing all three levels simultaneously in the same sample—remains an open avenue for research.

To these limitations of the literature analyzed are added the limitations of the present undertaking itself, discussed in Section 2, in particular the absence of a PRISMA-ScR procedure and the single-author selection process, which suggest that the convergences identified here should be interpreted as working hypotheses rather than definitive evidence.

Future research should therefore aim to: (a) directly validate, on ecologically valid samples, the three-level integrative model, through the simultaneous assessment of dispositional traits, maladaptive configurations, and criminogenic cognitions in the same participants; (b) expand cross-cultural comparative research, particularly in Eastern Europe, where the specific institutional and legislative context may shape personality profiles and criminogenic risk mechanisms; and (c) move from the level of global dimensions to that of facets and configural models, for greater predictive precision in recidivism risk assessment. Future work would also benefit from incorporating protective and developmental factors largely absent from the frameworks reviewed here—adverse childhood experiences, touched on only indirectly through Walters (2022), attachment style, and prosocial behavior—which may help explain individual variability in resilience against the dispositional and cognitive risk patterns identified above.

## 6. Conclusions

The five theoretical frameworks analyzed in this work—developed independently, within different research traditions and decades—converge on the same structural pattern when viewed together: a relatively constant dispositional predisposition (elevated aggression, low conscientiousness and honesty–humility) that does not act directly on behavior, but rather through dynamic mechanisms—self-efficacy, self-control, coping—that are, at least in principle, amenable to modification through intervention. The pattern was not derived from a formal statistical model but emerged as an interpretive synthesis of recurrent findings across heterogeneous studies, samples, and instruments that were not originally designed to communicate with one another. The value of the proposed integrative model lies precisely in this capacity to reveal a multilevel architecture of antisocial vulnerability where the fragmented literature showed only five parallel lines of research.

For the practice of psychological assessment in the prison setting, the direct implication is that profiling based exclusively on stable personality traits remains insufficient: the information of value for intervention lies at the level of the mechanisms linking predisposition to behavior, and it is precisely at this level that these mechanisms represent potentially modifiable targets relevant to correctional intervention. Directly testing this three-level architecture within a single design that assesses all components simultaneously in the same participants remains the necessary next step—a direction for which the recent series of studies on the Romanian prison population already provides a useful empirical foundation.

## Figures and Tables

**Table 1:** Distribution of the 109 sources across theoretical frameworks and source type.

Framework	Empirical Studies	Meta-Analyses/ Systematic Reviews	Theoretical/ Conceptual	Other (Instrument, Method, Book)	Total
FPI	6	0	1	1	8
Big Five	7	4	2	0	13
HEXACO	11	1	3	0	15
Dark Triad/Tetrad, psychopathy	10	10	7	2	29
Criminogenic cognitions	12	3	2	0	17
Cross-cutting/general/methodological	10	5	6	6	27
Total	56	23	21	9	109

Note. Classification by primary framework and predominant source type, coded by the author; sources addressing more than one framework (e.g., Dark Triad in relation to HEXACO) were assigned to the framework given greatest emphasis in the source. “Other” includes instrument manuals, review-methodology papers, and books.

**Table 2:** Predominant characteristics of the personality profile identified in offender populations in studies based on the FPI/FPI-R.

Psychological Dimension (FPI/FPI-R Construct)	Score Direction	Functional and Criminological Implications	Source	Participant Sample (*N*)
Aggressiveness	Elevated	Associated with violent and conflictual behaviors	Rada et al. (2025a)	*N* = 857
Emotional excitability/ irritability	Elevated	Intense reactions to frustration and stress	Rada et al. (2025a) (Emotionality scale; approximate correspondence to this dimension)	*N* = 857
Impulsivity and emotional instability	Elevated	Reduced behavioral and emotional control	Volf (2015)	*N* ≈ 230
Psychological strain/ nervousness	Elevated	Increased vulnerability to stress and maladaptive coping	Rada et al. (2025a)	*N* = 857
Social orientation	Reduced	Deficient social integration and reduced norm compliance	Hosser et al. (2008)	*N* = 775
Interpersonal integration	Reduced	Relational difficulties and lower cooperative behavior	Ille et al. (2005)	*N* = 128
Coping and adaptation to detention	Deficient	Difficulties adapting to penitentiary environment and institutional constraints	Volf (2015)	*N* ≈ 230

Note. Sources are given per row; N reported where available: Hesener and Hilse (1983), *N* = 166 (Adelsheim, *n* = 96; Hameln-Tündern, *n* = 70); Hosser et al. (2008), *N* = 775; Rada et al. (2025a), *N* = 857; Ille et al. (2005), *N* = 128; Volf (2015), *N* ≈ 230. Rows without an independently verifiable source are marked as such; see the paragraph below for further discussion of evidentiary status.

**Table 3:** Offense profile in the HEXACO model according to type of deviant/criminal behavior.

HEXACO Dimension *	Fraud/Deception	Violent Offenses	General Delinquency/Recidivism	Juvenile Delinquency	Impulsive Offenses	Primary Source(s)/Participant Sample *(N)*
Honesty–Humility	Very low ↓↓	Low ↓	Low ↓	Low ↓	Low ↓	Bader et al. (2025), *N* = 12,496; Rolison et al. (2013), *N* = 45 offenders (vs. 46 nonoffenders)
Conscientiousness	Medium–low ↓	Low ↓	Very low ↓↓	Low ↓	Very low ↓↓	Bader et al. (2025), *N* = 12,496; Međedović (2017), *N* = 256; Rolison et al. (2013), *N* = 45 offenders (vs. 46 nonoffenders)
Agreeableness	Low ↓	Very low ↓↓	Low ↓	Low ↓	Low ↓	Međedović (2017), *N* = 256; Rolison et al. (2013), *N* = 45 offenders (vs. 46 nonoffenders)
Emotionality	Low ↓	Mixed *	Low ↓	Low ↓	Low ↓	Međedović (2017), *N* = 256; Rolison et al. (2013), *N* = 45 offenders (vs. 46 nonoffenders) (see note below for contradictory findings)
Extraversion	Medium–high ↑	Low ↓	Low ↓	Variable ~	Variable ~	Rolison et al. (2013), *N* = 45 offenders (vs. 46 nonoffenders)

* **Note.** ↓ indicates a reduced level relative to the comparison group; ↓↓ indicates a markedly reduced level; ↑ indicates an elevated level; ~ indicates high variability or inconsistent findings across studies. Contradictory findings regarding Emotionality across studies (Međedović, 2017; Rolison et al., 2013), discussed in the text above. Note. Cells summarize directional findings (low/high relative to comparison groups); underlying studies use heterogeneous designs and metrics not reducible to a single effect size. *N* reported where available: Bader et al. (2025), *N* = 12,496; Međedović (2017), *N* = 256. Rolison et al. (2013), *N* = 45 offenders (vs. 46 nonoffenders).

## Data Availability

No new data were created or analyzed in this study. Data sharing is not applicable to this article.
